# Consumption of ultra-processed foods by foreign-born adults rise with increased levels of acculturation in the United States

**DOI:** 10.3389/fpubh.2025.1570832

**Published:** 2025-05-15

**Authors:** Jennifer J. Barb, Li Yang, Euridice Martinez-Steele, Ayaan Ahmed, Patricia V. Medina, E. Michael Valencia, Anna E. Roberts, Nicole Farmer, Gwenyth R. Wallen

**Affiliations:** ^1^Translational Biobehavioral and Health Disparities Branch, NIH Clinical Center, Bethesda, MD, United States; ^2^School of Public Health, Center for Epidemiological Studies in Health and Nutrition, University of São Paulo, São Paulo, Brazil

**Keywords:** acculturation, ultra-processed food consumption, UPF, standard American diet (SAD), NHANES, dietary acculturation

## Abstract

**Objective:**

Ultra-processed food (UPF) consumption has been linked to increased risks of negative health outcomes such as type 2 diabetes, obesity, and all-cause mortality. Some studies have shown an increase of UPF consumption with acculturation, whereas the influence of the Western diet on non-US born individuals is an area of increasing interest. The aim of this work was to investigate UPF consumption with an acculturation index and to assess whether there was an interaction of UPF consumption with race/ethnicity in non-US born adults.

**Methods:**

Dietary intake of non-US born adults participating in the National Health and Nutrition Examination Survey (NHANES) between 2011 and 2018 was assessed using day one of 24-h dietary recall, and the relative daily energy intake comprised of UPFs was used as the outcome of interest. The effect of acculturation assessed by an acculturation index (AcI) on UPF consumption was investigated after controlling for significant covariates. Complex survey sample regression analyses were used to evaluate the association between UPF consumption and AcI.

**Results:**

Population sample (*n* = 3,639) was comprised of non-US born adults (50% male) between 19 and 70 (43.1 ± 0.40) years of age. Of the adults assessed, 42.2% had lower AcI scores of 0 to 2, whereas 57.8% were more accultured (AcI scores of 3–5). Overall, dietary energy from UPFs was about 43.3%. When controlling for co-variates, acculturation was significantly associated with UPF consumption (*β* = 0.03, s.e. = 0.004, *p* < 0.001), meaning that for every unit increase in AcI score, there was a 3 % increase in mean UPF consumption. When the interaction between AcI and race/ethnicity was assessed, there was no difference in the impact of AcI on UPF consumption among race/ethnicity groups (*p* = 0.052).

**Conclusion:**

Our findings indicate that greater acculturation is associated with higher UPF consumption. Given the links between high UPF consumption and adverse health outcomes, targeted interventions that promote healthier dietary choices—while preserving culturally relevant eating habits—are essential to support the well-being of non-US born populations.

## Introduction

**Figure 1:**
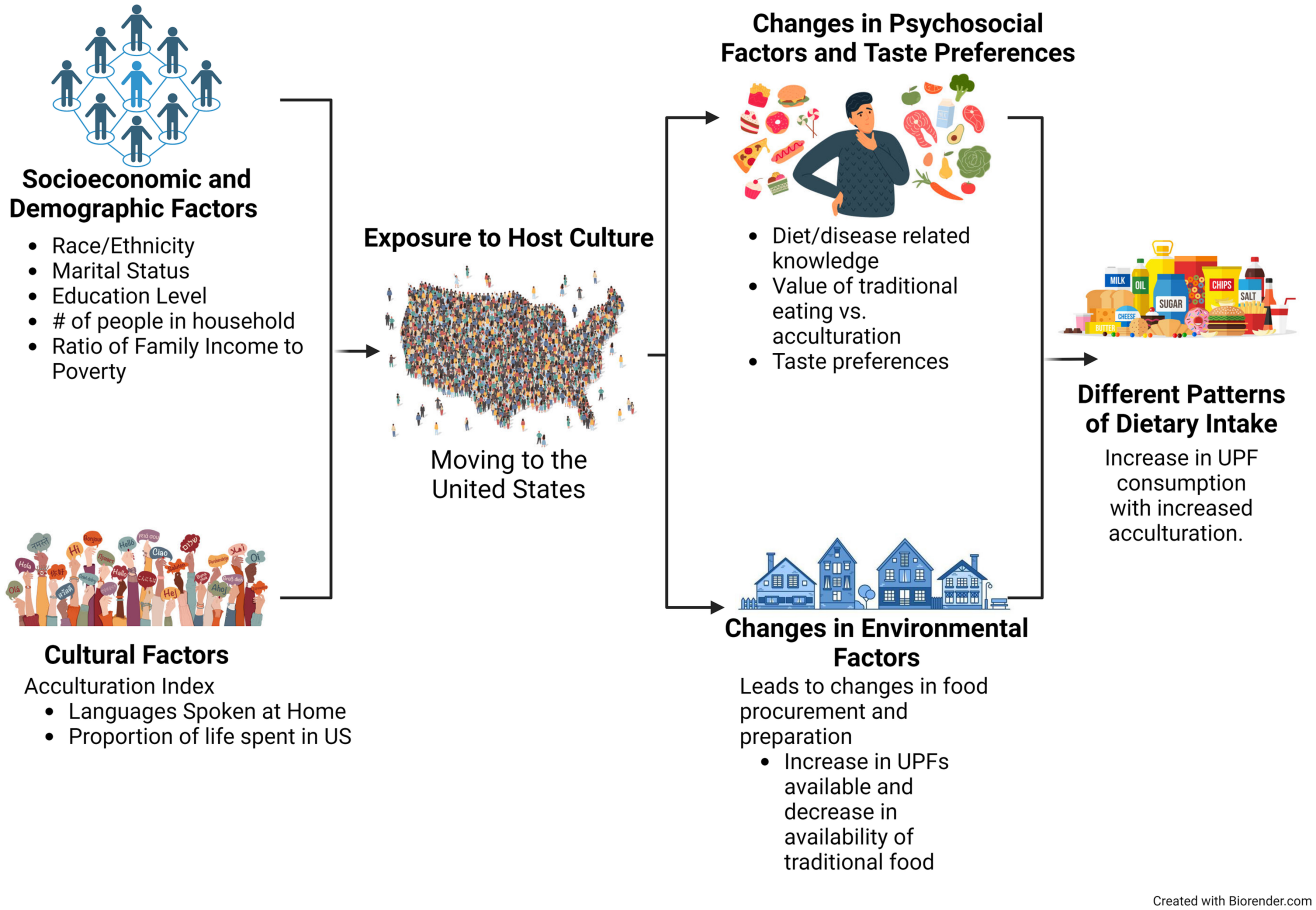
Dietary acculturation theoretic model. Associations of socioeconomic and cultural factors that may influence adaption and dietary acculturation to the Western diet. Created in BioRender.com.

Immigration often entails significant changes in lifestyle and environment as immigrants adjust to their new surroundings. One key aspect of this adjustment is the process of acculturation, wherein an immigrant’s values, attitudes, and behaviors evolve through continuous interaction with the ethnic majority of the host country (1). While acculturation can affect a wide range of changes, one notable area of change is dietary practices, which leads to what is known as *dietary acculturation*. This refers to the process through which immigrants may gradually abandon or alter their traditional dietary habits as they adopt new food practices from the host culture (2). Dietary acculturation is a multidimensional and dynamic process, influenced by factors at the individual level, as well as broader societal and systemic influences. The dietary acculturation model, proposed in 2002 by Satia-Abouta et al., was modified and adapted for this work in Figure 1, which shows how socioeconomic, demographic, and cultural factors are influenced by exposure to the host culture (2).

As of 2021, immigrants accounted for roughly 45 million (13.6%) people in the United States (US) population (3), a substantial proportion that has prompted growing interest in the dietary changes associated with immigration (2, 4, 5). Research has shown that a key dietary shift of immigrants is exposure to the standard American diet (SAD), a total dietary pattern (with multicultural variations) broadly characterized by excess consumption of calories from refined carbohydrates, processed foods, fatty meats, and added fats, often lacking in nutrient density (6). Furthermore, the SAD is typically lacking in a healthy consumption of fruits, vegetables, and whole grains- not meeting recommended dietary intake guidelines (6). A report found that, between the late 1950s and early 2000s, there was about a 761 kcal increase in average caloric intake, which has been associated with an increased average weight gain in Americans (6). In a recent 2018 study by Vangay et al., the authors highlight a significant impact of dietary changes on the human gut microbiome such that immigrants from Thailand exhibited a “Westernized” gut microbiome after migrating to the U.S., a change likely attributable to their altered diet (7). This work implicates the role of diet in not only altering cultural practices but also affecting physiological changes.

A growing body of research has begun to explore the SAD and the consumption of ultra-processed foods (UPFs) (8). The Nova classification framework was published by Carlos Monteiro in 2010 (9), providing a framework to further understand the impact of food processing on diet quality and health by classifying foods into four groups according to the nature, extent, and purpose of the industrial processing they undergo (10). The four groups are categorized as (1) unprocessed or minimally processed foods (MPF), (2) processed culinary ingredients, (3) processed foods, including products industrially manufactured with the addition of salt or sugar or other processed culinary ingredients to unprocessed/minimally processed foods, and (4) ultra-processed foods (UPF). UPFs include foods formulated with ingredients including sugar, oils, fats, salt, food substances, and cosmetic additives, mostly for exclusive industrial use that result from a series of industrial processes (10). Such foods are often nutritionally unbalanced and nutrient-deficient (11). A 2018 study, using data from the National Health and Nutrition Examination Survey (NHANES) between 2007 and 2012, revealed that Americans consumed 58.5% of their total caloric intake from UPFs (12). This trend has been linked to negative health outcomes (13–15), including increased risk of cardiovascular disease, obesity, all-cause mortality, type-2 diabetes, and cancer (14, 16). Additionally, a randomized-controlled trial by Hall et al. confirmed that consumption of UPFs led to weight gain, whereas the unprocessed food diet led to weight loss (17). A secondary analysis from the same study showed negative short-term human metabolomic changes in the individuals who consumed diets high in UPFs (18). These changes were observed in plasma and urine metabolites, which indicated changes in endogenous metabolism and recent dietary intake metabolism (18). Beyond health concerns, the increasing consumption of UPFs also has significant environmental implications, contributing to greenhouse gas emissions, water use, and land degradation due to the industrial production processes involved (19). In contrast, the production of MPFs, such as fruits, vegetables, nuts, and legumes, are associated with reduced greenhouse gas emissions per kilogram in Australia (20). While the US (21) is one of the largest consumers of UPFs, research is also showing concern about UPF consumption in other countries including, the UK (22), Canada (23), and Australia (24).

This study uses data from NHANES, (2011–2018) to explore the relationship between acculturation and UPF consumption among foreign-born adults in the US. The acculturation index (AcI) employed in this analysis assesses immigrants’ acculturation based on their primary language spoken at home and the proportion of life lived in the US. This work aims to examine how varying levels of acculturation correlate with patterns of UPF consumption across different ethnic and racial groups within the foreign-born population.

## Materials and methods

### Sample population

**Figure 2:**
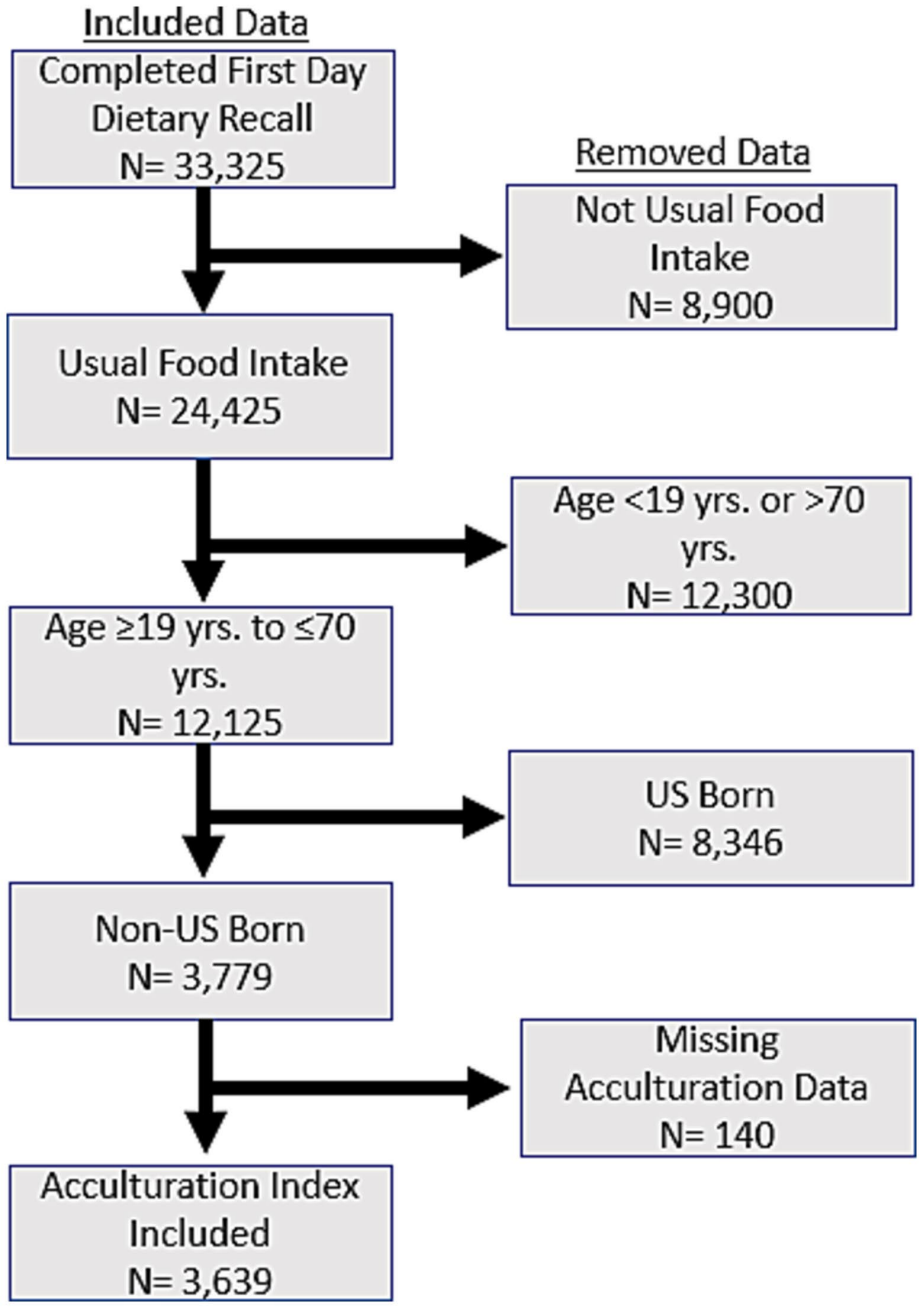
Data filtering process for the final analytical sample. Data filtering flow chart incorporating NHANES 2011–2018 survey data showing the generation of the final analytical sample size used for the analysis.

This study utilized data from four collection cycles of the National Health and Nutrition Examination Survey (NHANES), spanning from 2011 to 2018. The inclusion of these four cycles was aimed at increasing the sample size to enhance the statistical power of the analyses and to ensure conclusions were based on the most recent pre-pandemic evidence. The NHANES was conducted with the approval of the National Center for Health Statistics Research Ethics Review Board and adhered to local and institutional guidelines for human data collection. The dietary data used in this report were derived from the first 24-h dietary recall interview during an in-person interview in the Mobile Examination Center (MEC). A sample of 33,325 participants completed day 1 dietary recalls (Figure 2). To understand the impact of acculturation with UPF consumption, dietary recalls for participants reporting their ‘usual’ dietary intake were included, and any recalls not reported as ‘usual’ were removed. The ‘usual’ intake filter removed 8,900 respondents, leaving 24,425 for further filtering. Adults of the ages 19 through 70 were included, and individuals not meeting the age filter were removed (n = 12,300), leaving 12,125 respondents for further filtering. Because this work focuses only on foreign born individuals, those who reported being born in the US were removed (8,346), leaving 3,779 for further filtering. When incorporating the information from acculturation (using the ACQ files), there were 140 participants with missing acculturation information, and 3,639 is the sample size used in the final regression model.

### Dietary data and Nova food classification

The dietary data were derived from day 1 of the 24-h dietary recall based on previous research where only day 1 dietary data was assessed (25–27). Additionally, day 1 dietary data assessments were conducted during in person interviews at the mobile examination center, whereas day 2 assessments were conducted using phone interviews which could have created some discrepancies in collection. To understand the impact of acculturation respondents’ ‘usual’ dietary intake values, the respondent must have answered that the food recall was their ‘usual’ intake based on the question “*Was the amount of food that you ate yesterday much more than usual, usual, or much less than usual*?.” The dietary recall interview aspect of NHANES is also known as the What We Eat In America (WWEIA) Database (28). In WWEIA, each reported food and beverage is assigned an eight-digit food code (29, 30). In this system, food, energy, and nutrient values from the Food and Nutrient Database for Dietary Studies (FNDDS) are linked to the eight-digit food codes provided in WWEIA (29, 30). These energy and nutrient values are approximated based on the USDA National Nutrient Database for Standard Reference, also known as standard reference codes (SR codes) (29, 30).

As previously outlined in Steele et al. (31), all NHANES/WWEIA food codes were classified into four groups based on the Nova system and for foods judged as handmade recipes, the underlying SR codes were utilized for food classification. The percent of the Nova UPF daily caloric intake, derived using the ‘reference approach’ described by Steele et al., was the primary outcome of interest for this project.

### Acculturation index calculation

The AcI is comprised of the sum of a language score (LS) and a demographic score (DS) and is used as a score ranging from 0 (least accultured) to 5 (most accultured). The language score is categorized into 3 components, based on the language spoken at home as follows: English only (LS: 2 points), some English (LS: 1 point), or no English (LS: 0 points). Additionally, the demographics score is based on the proportion of life spent in the US and is categorized as follows: ≤10% (DS: 0), >10% to ≤25% (DS: 1), >25% to ≤50% (DS: 2), and > 50% of life spent in US (DS: 3). To calculate the proportion of life in the US, the midpoint of the range of years that the respondent reported having spent in the US, (<1: 0.5 years; 1 to <5: 3 years; 5 to <10: 7.5; 10 to <15: 12.5; 15 to <20: 25 years; 30 to <40: 35 years; 40 to <50: 45 years; ≥50: 50 years) is used as the numerator, which is divided by the age of the respondent at the time of the interview. The table below shows the midpoint used for each range:

**Table:** 

NHANES descriptor (years spent in US)	Midpoint in years
<1	0.5
1–<5	3
5–<10	7.5
10–<15	12.5
15–<20	17.5
20–<30	25
30–<40	35
40–<50	45
>50	50

### Covariates

Covariates included in the complex sampling analysis included age, sex, BMI (obtained from the BMX.XPT body measurement files), race/ethnicity, marital status, education level, household size, and income to poverty ratio. The income to poverty ratio, a valid indicator of social economic status, was categorized to three income subgroups: lowest (≤ 130% of the poverty threshold), middle (131–185%), and highest (> 185%). This ratio was calculated by dividing family income by the federal poverty guidelines specific to each survey year, which vary based on family size and geographic location (25). Marital status was dichotomized into married/living with a partner and not married. Race/ethnicity was coded into five groups as follows: Non-Hispanic White, Non-Hispanic Black, Asian, Hispanic and Other for anyone not fitting into the four race/ethnicity categories. Mexican American and Other Hispanic were grouped together as Hispanic. The education level was regrouped to three categories: high school or below, some college or an associate degree, and college graduates or above and any missing education information was not included in the analysis.

### Statistical analysis

SAS version 9.4 and JMP version 16 Statistical Computing Software (SAS Headquarters, Cary, NC) were used for analysis and visualization procedures for this study. The eight-year weights were calculated and used for all complex sample survey data analyses in this paper. Analyses were conducted using complete cases only. Appropriate descriptive statistics (frequencies and percentages for categorical variables and means and standard errors for continuous variables) were used to describe the whole sample. The PROC Surveyreg for complex sample survey data, Rao-Scott chi-square test, and SurveyCorrCov macro were used to assess the association between AcI, UPF, and demographic variables (age, sex, marital status, education level, BMI, household size, family income-to-poverty ratio, and race/ethnicity) (32). All variables that were significantly related to UPF from the bivariate analyses were entered into the final regression model to examine the adjusted effect of AcI on UPF. The interaction between AcI and race/ethnicity was tested to evaluate whether the impact of AcI on UPF differs across race/ethnicity groups. AcI is assessed in this analysis as an ordinal variable, where the higher ordinal AcI value, the more accultured an individual is considered. A *p-*value less than 0.05 was considered statistically significant.

## Results

### Sample population

This sample population was comprised of non-US born adults who had a mean age of 43.1 years, were 49.8% female, and had a mean BMI of 27.8 (Table 1). The sample population race/ethnicity is described as 48.3% Hispanic (including Mexican Americans and Other Hispanics), 25.9% Non-Hispanic (NH) Asian, 6.0% NH-Black, 16.5% NH-White, and 3.3% grouped into ‘Other’. The population included 26% not married individuals with an average family income-to-poverty ratio of 2.5, and 53.6% of the individuals were in the highest bin at ≥185% of poverty threshold. The average household size was 3.8 individuals per home.

**Table 1:** Demographics of study population.

*N* = 3,639 (unweighted)	Mean (SE) or n (%)
Age (years)	43.10 (0.40)
BMI	27.79 (0.16)
Sex	
Male	1814 (50.25%)
Female	1825 (49.75%)
Marital status	
Not Married	963 (26.06%)
Married/Living with Partner	2,624 (73.94%)
Number of Missing (n)	52
Education level	
High School or Below	1748 (45.21%)
Some College or Associate’s Degree	728 (20.94%)
College Graduate or Above	1,161 (33.85%)
Number of Missing (n)	2
Household Size	3.78 (0.05)
Number of Missing (n)	0
Income-to-Poverty Ratio % of poverty threshold	2.54 (0.08)
≤ 130% lowest	111 (32.88%)
131–185% middle	473 (13.49%)
>185% highest	1,642 (53.63%)
Number of Missing (n)	413
Race/Ethnicity	
Hispanic	1760 (48.29%)
Non-Hispanic White	187 (16.50%)
Non-Hispanic Black	261 (6.03%)
Non-Hispanic Asian	1,357 (25.90%)
Other	74 (3.27%)
Number of Missing (n)	0
Acculturation Index (AcI)	2.89 (0.047)
AcI = 0 n (%)	209 (5.743%)
AcI = 1 n (%)	459 (12.61%)
AcI = 2 n (%)	868 (23.85%)
AcI = 3 n (%)	899 (24.70%)
AcI = 4 n (%)	772 (21.21%)
AcI = 5 n (%)	432 (11.87%)
Total Energy (Kcal)	2076.02 (25.45)
Percent kcal of UPF	43.27% (0.007)

### Percentage of acculturation indices within race/ethnicity in non-US born individuals

**Figure 3:**
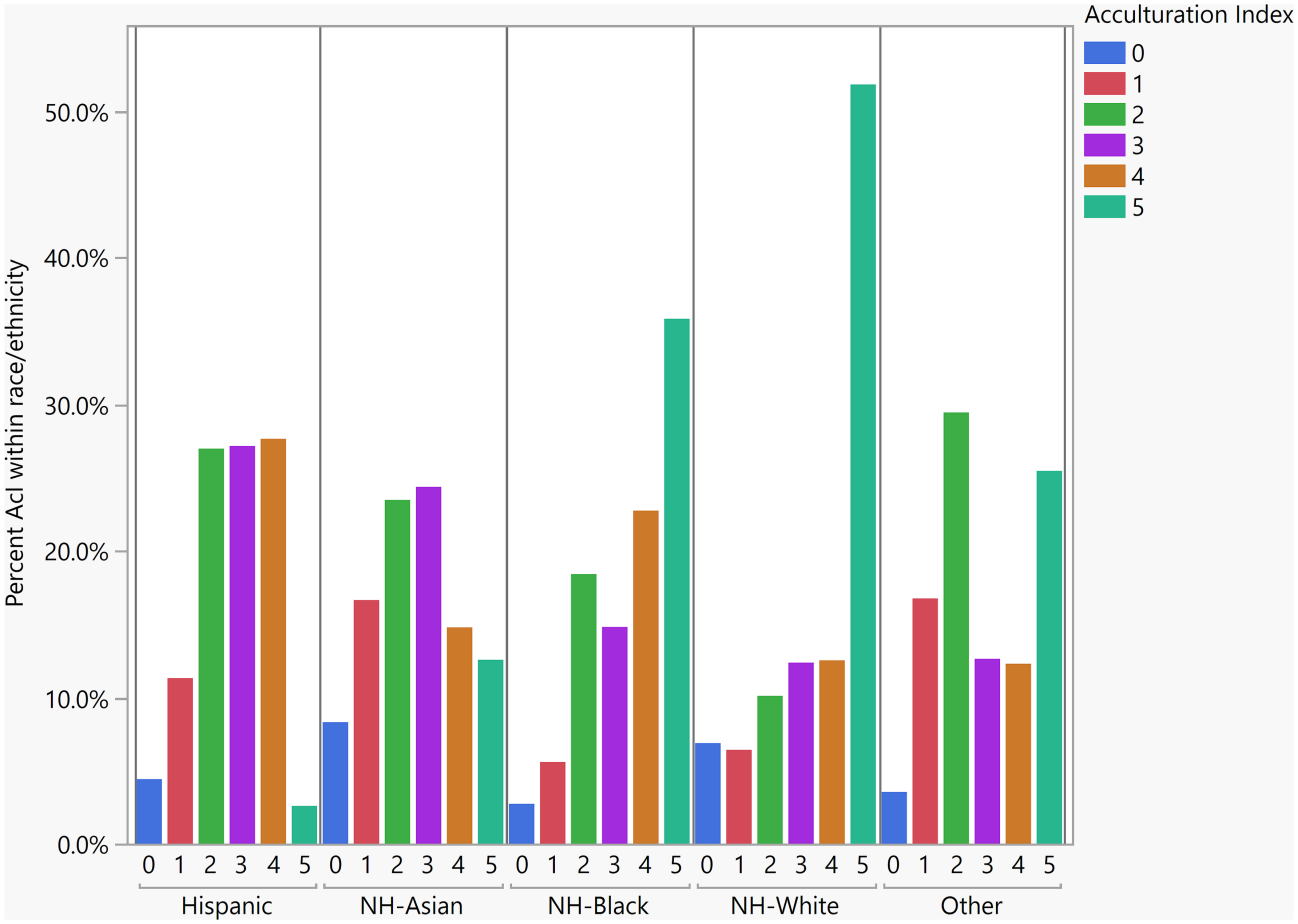
Distribution of acculturation index across race/ethnicities. Percent of the 6 levels of the acculturation index within each of the 5 race/ethnicity groups (weighted). NH-White, NH-Black and Other groups tended to be more acculturated (AcI value of 5) than were the Hispanic and NH-Asian groups. The acculturation variation across race/ethnicities was significant (*p* < 0.001).

Over the entire cohort, 42.16% of the sample had AcI values between 0 and 2, whereas 45.9% of the sample had acculturation values of 3 and 4, which is moderately acculturated. As previously mentioned, only 11.87% of the sample were the most acculturated with an AcI of 5 (Table 1). Furthermore, the smallest portion of the sample of 5.74% were the least acculturated (AcI = 0), which indicates that no English was spoken at home and less than 10% of their life has been lived in the US. Additionally, the percentage of acculturation within each race/ethnicity group was investigated to understand how acculturation was distributed across races and ethnicities among this foreign-born sample of individuals (Figure 3). Within the Hispanic group (combined Mexican American and Other Hispanic) race/ethnicity, only 2.58% were highly acculturated with an AcI of 5, while 4.44% of Hispanics were unacculturated with an AcI of 0. The majority of Hispanics were moderately acculturated with an AcI between 2 and 4, 26.95, 27.13 and 27.62%, respectively. When considering the NH-Asian group, 12.55% were the most acculturated and 8.29% were least accultured. The NH-Black group had the second highest percent of AcI 5 at 35.81%, while the NH-White group was the most acculturated at 51.8%. Overall, acculturation varied across each race/ethnicity groups [χ^2^ (20) = 346.61, *p* < 0.001].

### Percentage of diet from ultra-processed foods by race/ethnicity in non-US born individuals

**Figure 4:**
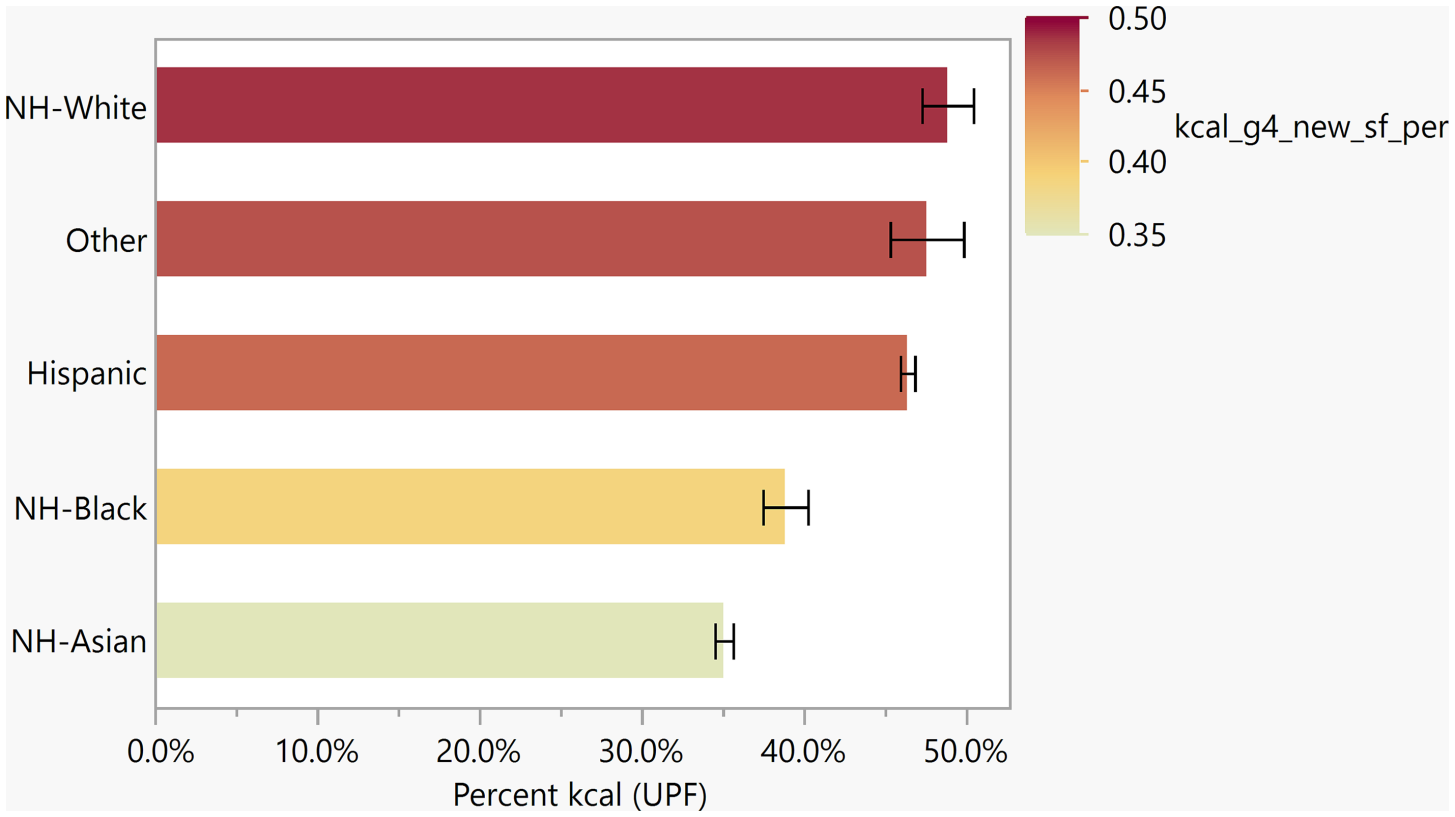
Percent of ultra-processed food intake across race/ethnicities. Average percent of kcal from UPF across 5 race/ethnicity groups. Percent across the race/ethnicity groups that consumed UPFs was highest among NH-Whites (47.9%) and lowest among NH-Asian (35.5%).Percent UPF consumed differed between the following race/ethnicities: NH-White vs. NH-Asian [*t*(62) = 5.82, *p* < 0.001] and vs. NH-Black [*t*(62) = 3.15, *p* = 0.025]; Hispanic vs. NH-Asian [*t*(62) = 11.78, *p* < 0.001] and vs. NH-Black [*t*(62) = 4.63, *p* < 0.001].

The average percent of UPF intake by race/ethnicity was investigated to understand how the consumption of UPFs differed by race/ethnicity. Overall, the average proportion of the diet across the entire sample of UPFs is 43.27% and when assessing this within race/ethnicity group, Non-Hispanic-White individuals had the highest percent of UPF intake (47.88%) compared to NH-Asians who had the lowest (35.48%; Figure 4). The race/ethnicity group of ‘Others’ which includes self-identified race/ethnicity groups not fitting into the other categories, showed the second largest proportion of UPF consumption of (43.22%). When the percent of UPF consumption was tested between races, NH-Whites and Hispanics consumed more of their total energy from UPFs than both NH-Asians and NH-Black, and more specifically, the percent of UPFs consumed differed between NH-White vs. NH-Asian [t(62) = 5.82, *p* < 0.001] and vs. NH-Black [t(62) = 3.15, *p* = 0.025]; Hispanic vs. NH-Asian [t(62) = 11.78, *p* < 0.001] and vs. NH-Black [t(62) = 4.63, *p* = 0.0002]. There were no significant differences observed between the ‘Other’ group compared to NH-White, NH-Asian or NH-Black, nor were there any significant differences observed between NH-White versus Hispanic and NH-Asian versus NH-Black.

### Association of acculturation and ultra-processed food consumption in non-US born individuals

**Figure 5:**
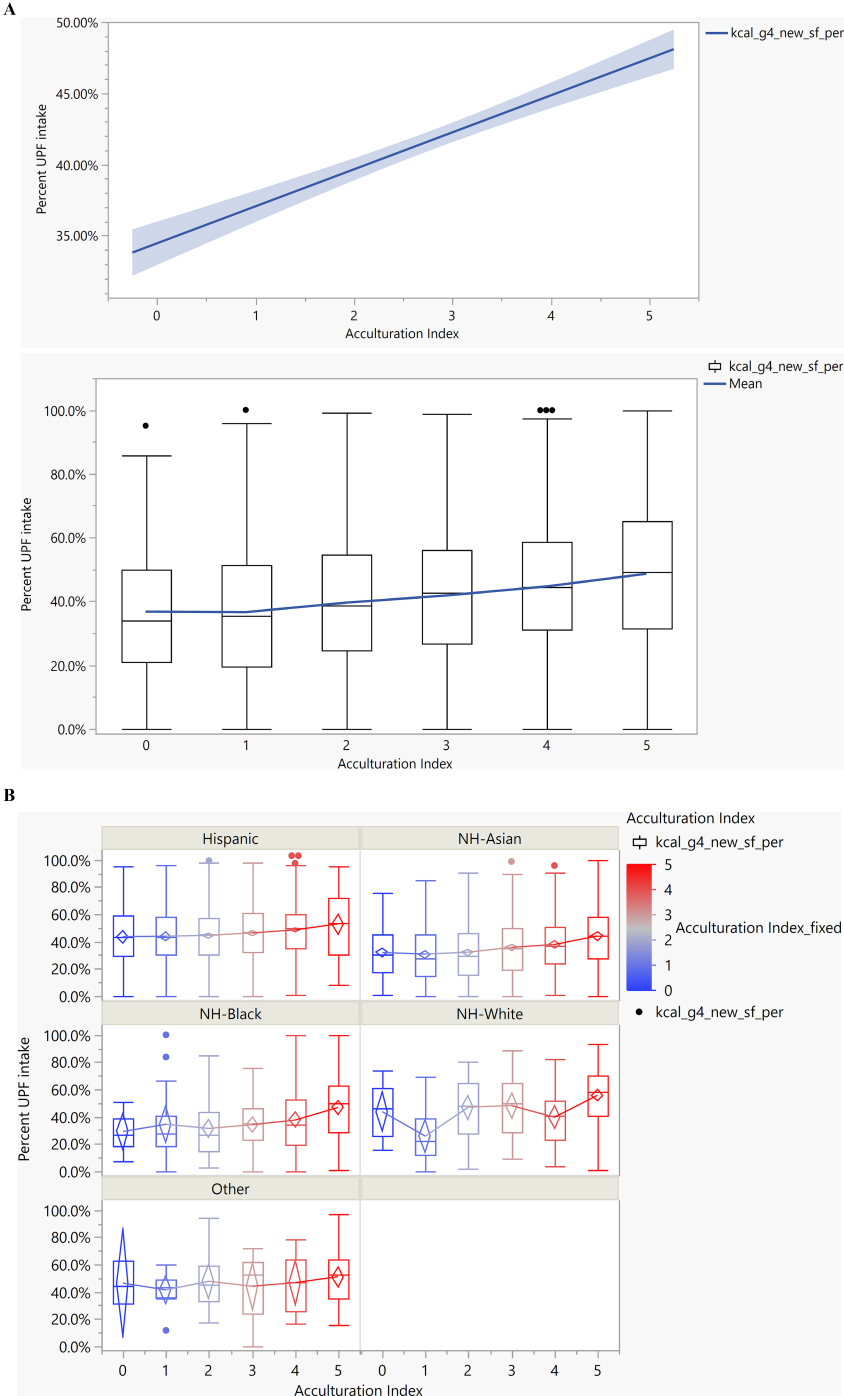
The association between acculturation and ultra-processed food consumption. **(A)** Average percent of energy from UPF across AcI for entire population. Significant linear trend as AcI increases, so does UPF consumption (*β* = 0.03, s.e. = 0.004, *p* < 0.001). **(B)** Percent of energy from UPF (y-axis) and AcI (x-axis) by race/ethnicity. There is no significant difference in the impact of AcI on the percent of UPF consumed across race/ethnicities (*p* = 0.052). Blue to red color gradient indicates the AcI levels.

The impact of acculturation on UPF consumption was investigated while controlling for age, BMI, race/ethnicity, and sex (Figure 5; Table 2). Covariates that were either not significantly related to UPF consumption in bivariate analyses including household size (*p* = 0.064), income-to-poverty ratio (*p* = 0.571), and sex (*p* = 0.498), or that became non-significant in the models after controlling for other covariates including marital status and education status, were not included in the final model.

**Table 2:** Final regression model testing the impact of acculturation on UPF consumption.

	Estimate	Standard error	t value	Pr > |t|
Race/Ethnicity (ref: Hispanic)				
Non-Hispanic White	−0.017	0.018	−0.92	0.361
Non-Hispanic Asian	−0.097	0.010	−9.22	<0.001
Non-Hispanic Black	−0.1063	0.016	−6.52	<0.001
Other	−0.037	0.039	−0.95	0.347
Sex (ref: Male)				
Female	0.002	0.009	0.23	0.822
Acculturation Index	0.032	0.004	8.45	<0.001
BMI	0.003	0.001	2.98	0.004
Age	−0.003	0.000	−6.52	<0.001

In this sample of non-US born individuals, as acculturation increased, the percent of calories consumed from UPFs also increased linearly (*β* = 0.03, s.e. = 0.004, *p* < 0.001; Figure 5A; i.e., for every unit increase in AcI score, there was a 3 % increase in mean UPF consumption). Furthermore, when investigating the interaction between race/ethnicity and AcI on UPF consumption, while controlling for BMI and age, there was no significant interaction (*p* = 0.052). Thus, overall, the impact of AcI on UPF consumption did not differ across race/ethnicity groups (Figure 5B).

## Discussion

In recent years, public health research has increasingly focused on the associations between diets high in UPFs and various health risks, especially in the US, where a large proportion of the SAD is comprised of UPFs (33). Given the high proportion of immigrants in the US (3, 4), the impact of acculturation on UPF consumption is an area of research in need of further attention. Over the last few years, studies have begun to explore the relationship between acculturation and UPF consumption (4, 13, 34), and this work extends that research by investigating foreign born individuals from NHANES 2011–2018 with examining associations between UPF consumption, socioeconomic status and acculturation using a modified AcI.

We include a model of dietary acculturation, which is a theoretical model adapted from Satia-Abouta et al. (2). This model accounts for socioeconomic, demographic, and cultural factors. Immigrants are often introduced to new dietary options and habits that may not be common in their countries of origin (2). In the US, this exposure to the host culture may lead to increased consumption of UPFs for individuals from another country, where UPFs are not as readily available. The environment in which an individual resides can heavily influence food choices based on the ease of availability, food preparation-related time constraints, and lack of access to fresh foods. Psychosocial factors influence dietary patterns when individuals are exposed to the social norms and attitudes of the host culture, as is the case in the US, where UPFs are regularly consumed. An individual’s process of acculturation is shaped by their social, demographic, and cultural background, which in turn impacts the degree to which they adopt host cultural practices (Figure 1).

The AcI metric used in this analysis is based on the one proposed by Pachipala et al. in 2022, which found an increase in UPF consumption with acculturation among Asian Americans (4). However, there are key differences between the two studies. First, the AcI in this study was calculated using two metrics, whereas Pichipala et al. employed three. Additionally, the current study focuses on all foreign-born race/ethnic groups, not just Asian Americans. A significant distinction lies in how the AcI composite was calculated; this study incorporates the proportion of life spent in the US (years in the US/age of individual) rather than the absolute number of years spent in the US, as used by Pachipala et al.

Similarly, Steele et al. (34) using NHANES data from 2011 to 2016, found that foreign-born adults who spent more time in the US consumed more UPFs. They also observed that individuals who spoke English at home exhibited similar patterns. However, Steele’s study differs from the current study by including both foreign born and native-born individuals. Other studies have also examined UPF consumption and birth status, such as one by Sharkey et al., which demonstrated that Mexican-born and US-born women living along the Texas-Mexico border consumed significantly more sugar-sweetened beverages and fast food meals than others (35). Furthermore, other studies by Creighton et al. (36) and Peters et al. (37), explored relationships between acculturation and diet, focusing on obesity and the gut microbiome. Specifically, the study by Peters et al., found that US dietary acculturation may result in loss of native gut microbial species in immigrants. A similar loss of microbial diversity was observed by Vangay et al. in 2018, among individuals who migrated to the US from Hmong and Karen (Thailand) communities (7). This loss of gut microbial diversity may be linked to increased UPF consumption as immigrants experience greater dietary acculturation. Along with these dietary changes, immigrants often develop a higher prevalence of co-morbidities associated with metabolic syndrome and this intersection of dietary acculturation, gut microbiome health, and metabolic health remains an area of research in need of more attention. Our work showed a significant positive linear relationship between the AcI metric and the proportion of energy from UPFs (Figure 5A), which can be interpreted as: for each unit increase in the AcI, there was a 3 % increase in mean UPF-consumption. As just discussed, Steele (34) and Pachipala (4), both corroborate these findings.

Socioeconomic status (SES) may play a significant role in increased consumption of UPFs. Individuals of lower SES may be more likely to consume diets higher in UPFs due to factors such as lower cost, greater availability and food insecurity. Additionally, living in areas like ‘food deserts’, which are often found in lower-income neighborhoods, can contribute to this trend (38). In fact, UPF consumption in the US has been linked to lower educational attainment, a lower income-to-poverty ratio, as well as immigrant status (39). Our nationally representative NHANES cohort of foreign-born individuals, showed no association of income to poverty ratio with UPF consumption (*p* = 0.571). The average income to poverty ratio in this cohort of foreign-born individuals is 2.54, indicating that the household income is 2.5 times higher than the poverty level, and 53% of the sample is in the highest percent poverty threshold of > 185%. This may explain why no association was observed between income and UPF consumption, as the smaller sample size could limit the power to detect such a relationship.

While this work shows results on a focused sample population of foreign-born individuals and the association with UPF consumption using a modified AcI, some limitations should be addressed. The dietary recalls used assume that the intake is a typical day of eating, and this was controlled for by using only recalls where the user answered, “usual intake,” though some variability in recall and intake reporting may exist, which will unlikely affect the studied associations. This study focused only on non-US-born individuals and therefore the generalizability to a broader population is limited. Further research with a larger sample of non-US-born and US-born populations from immigrated parents may help to build upon these findings. Also related to the Nova coding, since the initial dietary intake assessments were not collected for the purpose of Nova categorization, some assumptions had to be made, and therefore, the categorization could be prone to some errors, which have been discussed in the previous publication (31). Additionally, the calculated percentage of energy contribution from UPFs may underrepresent foods with little energy content; however, this is unlikely to substantially affect the associations explored in the study. This report does not infer any causality and only highlights the associations between acculturation and UPF consumption. Additional research on this topic is warranted and perhaps a cohort study of non-US born immigrants studying the evolution of UPF consumption, AcI over time and its impact on gut microbiota and health outcomes would be suitable to expand this area of research.

This analysis shows an increase in UPF consumption with acculturation in a sample of non-US born individuals using NHANES survey data from the years of 2011–2018, and the findings here are corroborated by previous research on this topic. While the SAD is comprised of a large percentage of UPFs, it is crucial to implement educational practices that enhance food labeling, particularly by indicating the level of food processing. Investigating the impact of acculturation on UPF consumption among foreign-born individuals in the US allows for thoughtful consideration on developing culturally tailored strategies to improve dietary habits across diverse populations. Additionally, intervention strategies should be considered for non-U.S.-born individuals, promoting healthy food practices that incorporate traditional diets from their countries of origin. This approach would support the integration of diverse cultural foodways into the U.S. melting pot, fostering healthier eating behaviors across immigrant communities. In fact, Olstad et al., described an erosion of cultural food practices of self-reported Indigenous people in Canada whereas, diet quality scores were lower and mean UPF intake were higher when compared to all other six racial/ethnic minority groups (40). Of course, however, it is important to note that knowledge related to health and nutrition may also play a key factor in dietary acculturation. Some individuals may adopt or modify their dietary habits based on health recommendations prevalent in the US, leading to changes in food choices and cooking methods. Overall, the relationship between acculturation and UPF consumption underscores the complex interplay between cultural, economic, social, and environmental factors in shaping dietary habits and health outcomes. Developing community-based, culturally competent, and relevant dietary interventions that capitalize on the healthy behaviors that immigrant populations bring to the US may serve as protective factors despite increased exposure to the Standard American Diet and UPFs.

## Conclusion

This analysis shows an increase in UPF consumption with acculturation in this sample of non-US born individuals using NHANES survey data from the years of 2011–2018, and the findings here are corroborated with previous research on this topic. While the SAD is comprised of a large percentage of UPFs, educational practices should be considered and implemented, such as better food labeling indicating processing levels of certain foods. Interventional strategies should be considered for non-US born individuals to promote culturally-based diets that encourage healthy eating behaviors from their countries of origin. It is important to note that knowledge related to health and nutrition may also play a key factor in dietary acculturation. Some individuals may adopt or modify their dietary habits based on health recommendations prevalent in the US, leading to changes in food choices and cooking methods. Overall, the relationship between acculturation and UPF consumption underscores the complex interplay between cultural, economic, social, and environmental factors in shaping dietary habits and health outcomes. Developing community-based, culturally competent, and relevant dietary interventions that capitalize on the healthy behaviors that immigrant populations bring to the US may serve as protective factors despite increased exposure to the Standard American Diet and UPFs.

## Data Availability

Publicly available datasets were analyzed in this study. This data can be found at: https://wwwn.cdc.gov/nchs/nhanes/.
